# The chick pallium displays divergent expression patterns of chick orthologues of mammalian neocortical deep layer-specific genes

**DOI:** 10.1038/s41598-019-56960-4

**Published:** 2019-12-31

**Authors:** Toshiyuki Fujita, Naoya Aoki, Eiko Fujita, Toshiya Matsushima, Koichi J. Homma, Shinji Yamaguchi

**Affiliations:** 1 Faculty of Pharmaceutical Sciences, Department of Life and Health Sciences, Teikyo University, 2-11-1 Kaga, Itabashi-ku, Tokyo 173-8605 Japan; 2 Department of Biology, Faculty of Science, Hokkaido University, Hokkaido, 060-0810 Japan

**Keywords:** Neuroscience, Evolution

## Abstract

The avian pallium is organised into clusters of neurons and does not have layered structures such as those seen in the mammalian neocortex. The evolutionary relationship between sub-regions of avian pallium and layers of mammalian neocortex remains unclear. One hypothesis, based on the similarities in neural connections of the motor output neurons that project to sub-pallial targets, proposed the cell-type homology between brainstem projection neurons in neocortex layers 5 or 6 (L5/6) and those in the avian arcopallium. Recent studies have suggested that gene expression patterns are associated with neural connection patterns, which supports the cell-type homology hypothesis. However, a limited number of genes were used in these studies. Here, we showed that chick orthologues of mammalian L5/6-specific genes, *nuclear receptor subfamily 4 group A member 2* and *connective tissue growth factor*, were strongly expressed in the arcopallium. However, other chick orthologues of L5/6-specific genes were primarily expressed in regions other than the arcopallium. Our results do not fully support the cell-type homology hypothesis. This suggests that the cell types of brainstem projection neurons are not conserved between the avian arcopallium and the mammalian neocortex L5/6. Our findings may help understand the evolution of pallium between birds and mammals.

## Introduction

The organisation of the avian telencephalic pallium differs considerably from that of the mammalian neocortex, which forms the neural basis of cognitive abilities in mammals^1,2^. Whilst large areas of the mammalian cortex exhibit a six-layered structure, large parts of the avian pallium are not laminated, but are instead organised into clusters of neurons (nuclei)^2^. The large masses of neurons make up the dorsal ventricular ridge (DVR), which is a highly elaborate pallial structure. The DVR consists of the mesopallium, nidopallium, and arcopallium. The difference in organisation has raised questions with regard to the evolutionary relationship between sub-regions of avian pallium and layers of mammalian neocortex^3,4^.

Although the overall organisation of the avian DVR and mammalian neocortex are different, the fundamental neural connections in sensory input and motor output pathways are common to mammals and birds^5,6^. For example, in mammals, layer 5 (L5) projection neurons of the motor cortex extend to the brainstem and spinal cord, and layer 6 (L6) projection neurons primarily project to the thalamus^7^. In the bird DVR, neurons in the anterior, dorsal, and intermediate parts of the arcopallium also project to the brainstem and rostral spinal cord^8–11^. This evidence indicates that the neural connections in the cell populations of the bird arcopallium and mammalian L5/6 similarly project to sub-pallial targets, such as brainstem and premotor areas (motor output). It has been proposed that brainstem projection neurons in the avian arcopallium are homologous to brainstem projection neurons in the neocortex L5/6 (cell-type homology hypothesis)^5,6,12^. Concomitantly, a study has shown that six chick orthologues of neocortex L5/6 markers were strongly expressed in the arcopallium, which is the motor output region of the bird DVR^13^. This suggests that gene expression patterns reflect the neural connection patterns of motor output projections to sub-pallial targets between the DVR and neocortex. Whilst their results therefore support the cell-type homology hypothesis, a limited number of genes was used. A recent largescale transcriptomic analysis revealed a mostly divergent pattern in pallial compartments between chicken and mouse^14,15^, suggesting the possibility that the expression of chick orthologues of L5/6 genes do not fully support the cell-type homology hypothesis.

In this study, we examined the expression of more chick orthologues of L5/6 genes and tested whether their expression patterns supported the cell-type homology hypothesis. If cell types are conserved between the brainstem projection neurons in the mammalian L5/6 and the avian arcopallium, most of the chick orthologues of L5/6-specific genes should be selectively expressed in the arcopallium. We used chick orthologues of mammalian neocortical L5/6 markers and performed *in situ* hybridisation in the chick telencephalon. We found *nuclear receptor subfamily 4 group A member 2* (*NR4A2*) and *connective tissue growth factor* (*CTGF*) expression in the arcopallium which reflected the neural connection patterns between avian DVR and mammalian neocortex. However, the gene expression patterns of the other four did not. Their major expression regions were outside of the arcopallium, which shows that the expression patterns of the four genes did not reflect the neural connections in terms of motor output. Our results on the expression patterns of chick orthologues of L5/6 genes do not fully support the cell-type homology hypothesis, which suggests that the cell types of brainstem projection neurons are not conserved between the avian arcopallium and the neocortex L5/6.

## Results

### Selection of the mammalian neocortical layer-specific marker genes

We selected six chick orthologues that have been shown to be mammalian neocortical L5/6-specific or -selective markers^16–20^, i.e. *NR4A2*, *CTGF*, *neurofilament heavy polypeptide* (*NEFH*), *thymocyte selection-associated high mobility group box* (*TOX*), *chicken ovalbumin upstream promoter transcription factor interacting protein 2* (*CTIP2*) and *forkhead box protein P2* (*FOXP2*). *NR4A2* and *CTGF* expressed in neocortical L6 specifically, *NEFH* and *TOX* expressed in L5 specifically, and *Ctip2* expressed in both L5 and L6, and *Foxp2* was expressed in the L6 selectively in mice neocortex^16–20^ (Table 1). The six orthologues exhibited the following sequence similarities between chicks and mice: *NR4A2*: protein 94.6% and DNA 85.3%; *CTGF*: protein 92.6% and DNA 84.3%; *NEFH*: protein 62.6% and DNA 66.9%; *TOX*: protein 89.6% and DNA 82.1%; *CTIP2*: protein 84.4% and DNA 76.2%; and *FOXP2*: protein 93.8% and DNA 87.5%. Then, we confirmed their expression pattern by referring to the data in the Allen Mouse Brain Atlas^21–23^ (http://www.brain-map.org, Table 1). When we selected genes from Allen Brain Atlas, we did not focus on the function of genes. Regardless of the functions, we selected genes expressed in deep layers of neocortex selectively, with less expression in other parts of pallium, especially in the pallial amygdala. We performed *in situ* hybridisation and analysed the expression pattern of the chick orthologues in the chick brains.

**Table 1:** Overview of markers of cortical layers 5 and 6 in this study.

Accession number	Gene symbol	Gene name	Expression layer in cortex	References	Experiment number at Allen Brain Atlas
CR522946	*NR4A2*	Nuclear receptor subfamily 4, group A, member 2	Layer 6 (Subplate)	Watakabe, *et al*.^18,19^, Molyneaux, *et al*.^16^	732
NM_204274	*CTGF*	Connective tissue growth factor	Layer 6 (Subplate)	Heuer, *et al*.^17^	1183
XM_415310	*NEFH*	Neurofilament, heavy polypeptide	Layer 5	Molyneaux, *et al*.^16^	74512048
XM_015282673	*TOX*	Thymocyte selection-associated high mobility group box	Layer 5	Artegiani, *et al*.^20^	71670691
XM_003641410	*CTIP2*	Chicken ovalbumin upstream promoter transcription factor interacting protein 2	Layer 5 and 6	Molyneaux, *et al*.^16^	74990505
NM_001318413	*FOXP2*	Forkhead box protein P2	Layer 6	Molyneaux, *et al*.^16^	72079884

### NR4A2 expression in the telencephalon of chicks

**Figure 1:**
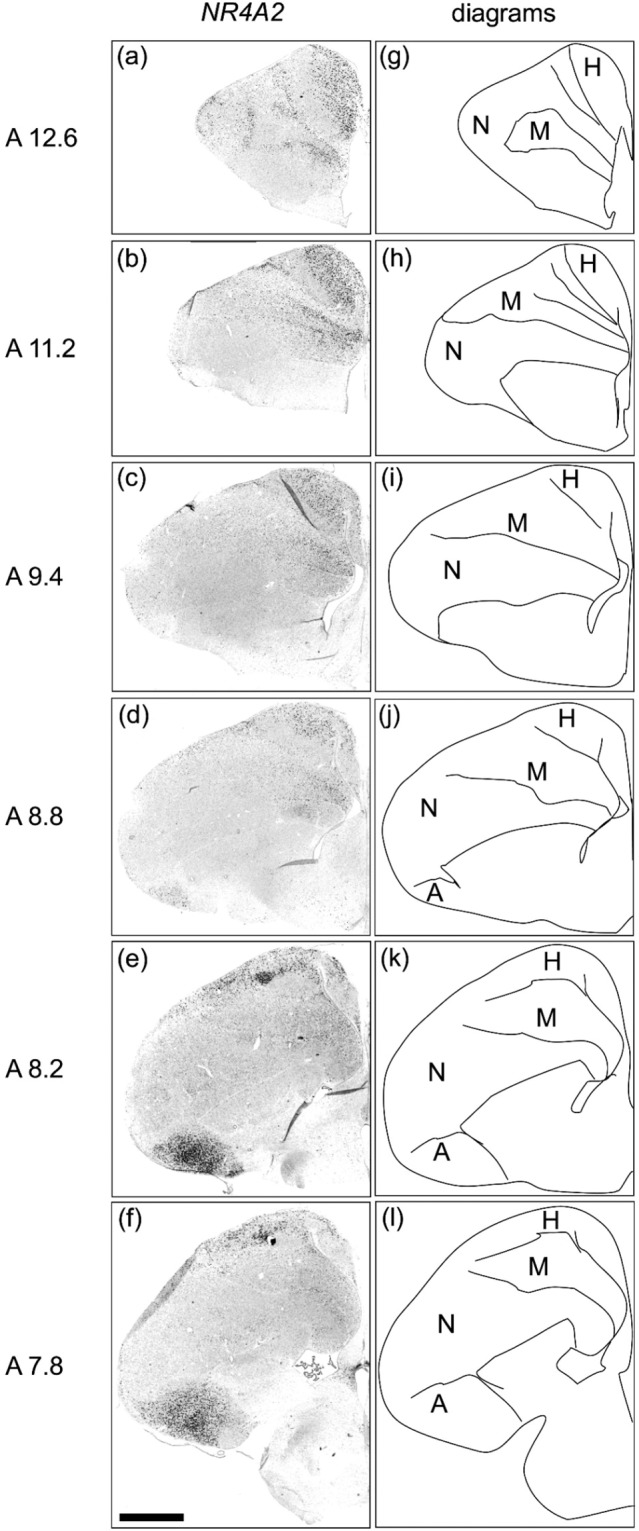
*In situ* hybridisation of *NR4A2* in P1 chick brains. DIG-labelled RNA antisense (**a**–**f**) *NR4A2* probe was used for *in situ* hybridisation in P1 chick brain coronal sections. For *NR4A2*, sections of two chicks were analysed, and representative images of chick brain sections are shown. (**g**–**l**) Diagrams of coronal sections are shown on the right panels. The levels of the sections (A12.6 to A7.8) correspond to those of the chick atlas by Kuenzel and Masson^50^. A, arcopallium; H, hyperpallium; M, mesopallium; N, nidopallium. Scale bar = 2.5 mm.

We performed *in situ* hybridisation using *NR4A2* as the mammalian neocortical layer 6b-specific marker in post-hatched day-1 (P1) naive chick brains. Strong signals were detected in the hyperpallium (Fig. 1a–f,A12.6-A7.8) and arcopallium (Fig. 1d–f,A8.8-A7.8). In addition, signals were detected in the mesopallium (Fig. 1a–d,A12.6-A8.8). Previous studies have detected *NR4A2* expression in the hyperpallium and mesopallium in embryonic chicks^24,25^.

### CTGF expression in the telencephalon of chicks

**Figure 2:**
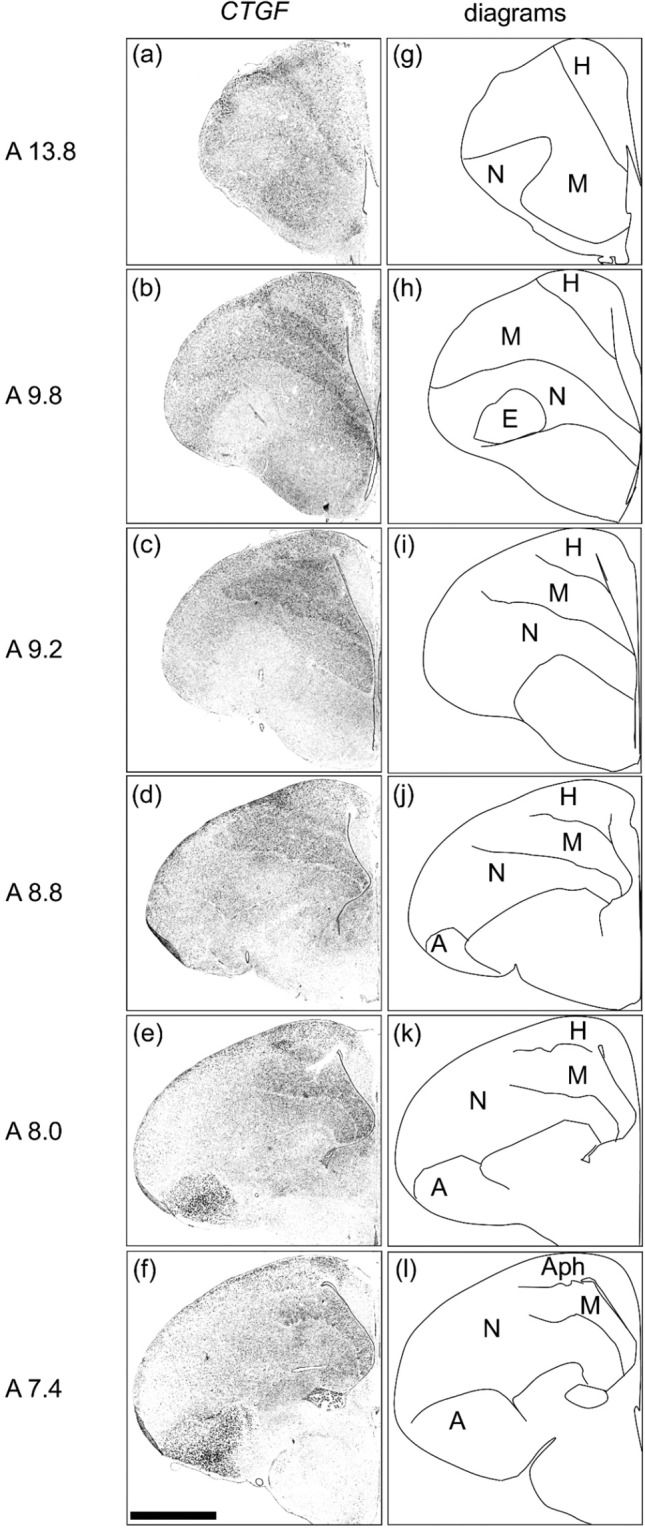
*In situ* hybridisation of *CTGF* in P1 chick brains. DIG-labelled RNA antisense (**a**–**f**) *CTGF* probe was used for *in situ* hybridisation in P1 chick brain coronal sections. For *CTGF*, sections of three chicks were analysed and representative images of two chick brain sections are shown. (**g**–**l**) Diagrams of coronal sections are shown on the right panels. The levels of the sections (A13.8 to A7.4) correspond to those of the chick atlas by Kuenzel and Masson^50^. A, arcopallium; Aph, area parahippocampalis; E, entopallium; H, hyperpallium; M, mesopallium; N, nidopallium. Scale bar = 2.5 mm.

Strong signals were detected in the mesopallium (Fig. 2a–f,A13.8-A7.4) and arcopallium (Fig. 2d–f,A8.8-A7.4). In addition, signals were detected in the hyperpallium (Fig. 2a–e,A13.8-A8.0) and parahippocampal area (APH) (Fig. 2f,A7.4). To the best of our knowledge, this is first time that *CTGF* expression in the arcopallium has been reported. *CTGF* expression in the hyperpallium and mesopallium has been described by Wang *et al*.^24^.

**Figure 3:**
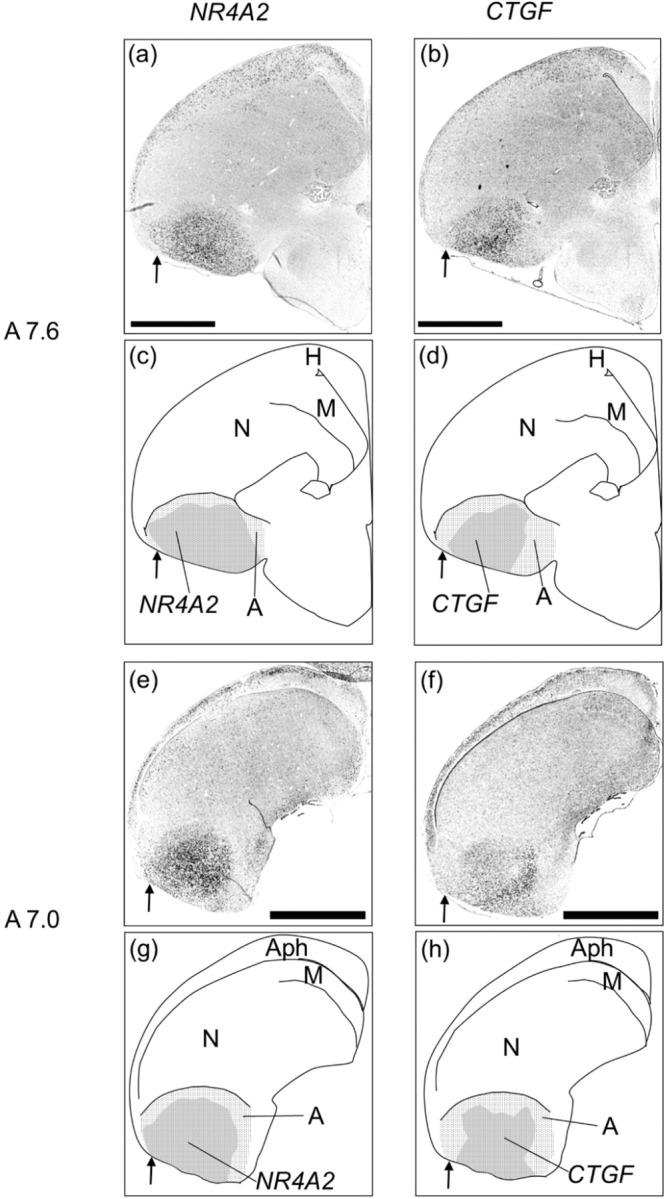
*In situ* hybridisation of *NR4A2* and *CTGF* in P1 chick brains using neighboring sections. *In situ* hybridisation using DIG-labeled RNA antisense *NR4A2* (**a**,**e**) and *CTGF* (**b**,**f**) probes with P1 chick brain coronal sections were shown, respectively. Panels (c), (d), (g), and (h) indicated the diagrams of the panels of the (a), (b), (e), and (f), respectively. The shaded regions, (**c**,**g**) for *NR4A2* and (**d**,**h**) for *CTGF*, indicated the regions of signal detected in the arcopallium (dot pattern regions), respectively. Arrows indicated the area of the lateral arcopallium (**a**–**h**). The levels of the sections (A7.6 to A7.0) correspond to those of the chick atlas by Kuenzel and Masson^50^. A, arcopallium; Aph, area parahippocampalis; H, hyperpallium; M, mesopallium; N, nidopallium. Scale bar = 2.5 mm.

We also found different expression patterns of *NR4A2* and *CTGF* in the arcopallium (Fig. 3,A7.6 and A7.0). *NR4A2* was expressed almost ubiquitously in the lateral, ventral, and intermediate arcopallium, while *CTGF* was highly expressed in the medial and ventral arcopallium, but not in the lateral arcopallium. Neither *NR4A2* nor *CTGF* was expressed in the dorsal arcopallium.

### NEFH expression in the telencephalon of chicks

**Figure 4:**
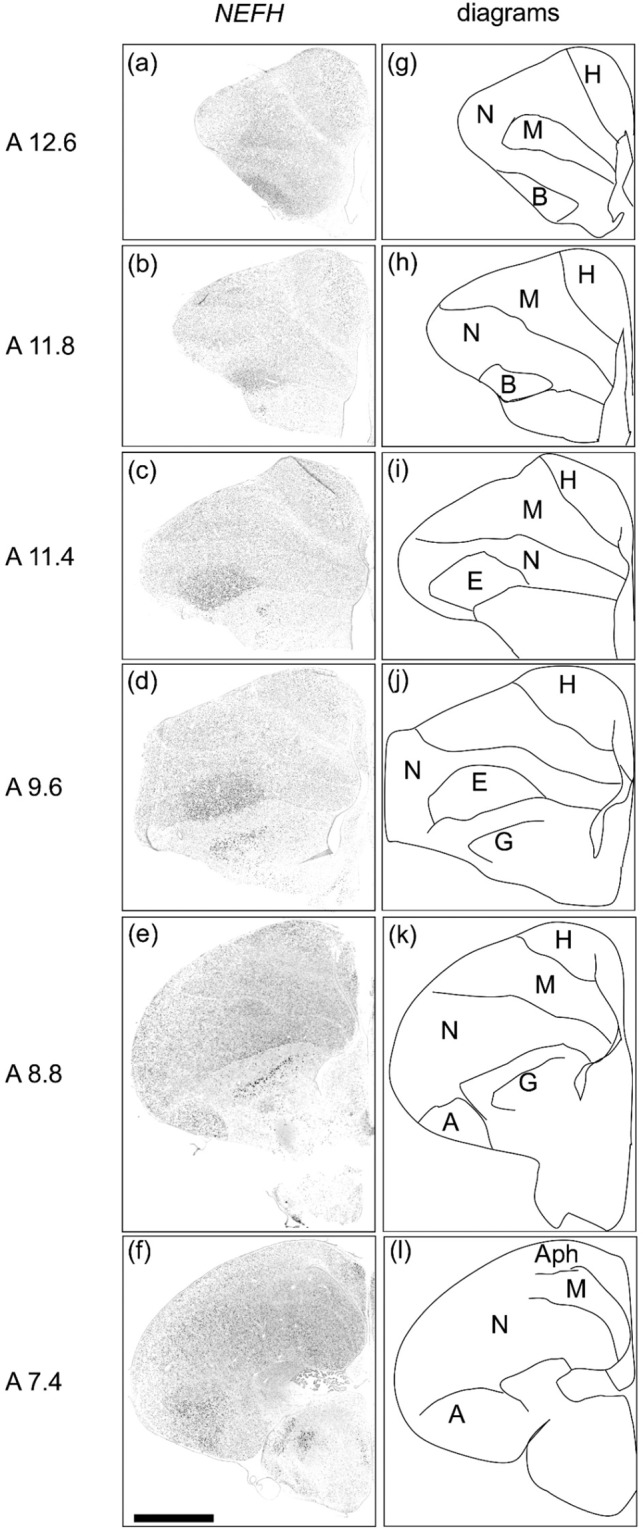
*In situ* hybridisation of *NEFH* in P1 chick brains. DIG-labelled RNA antisense (**a**–**f**) *NEFH* probe was used for *in situ* hybridisation in P1 chick brain coronal sections. For *NEFH*, sections of four chicks were analysed and representative images of three chick brain sections are shown. (**g**–**l**) Diagrams of coronal sections are shown on the right panels. The levels of the sections (A12.6 to A7.4) correspond to those of the chick atlas by Kuenzel and Masson^50^. A, arcopallium; Aph, area parahippocampalis; B, basorostralis; E, entopallium; G, globus pallidus; H, hyperpallium; M, mesopallium; N, nidopallium. Scale bar = 2.5 mm.

Strong signals were detected in the basorostralis (Fig. 4a,A12.6,b,A11.8), entopallium (Fig. 4c,A11.4,d,A9.6), and globus pallidus (Fig. 4d,A9.6,e,A8.8). Relatively weak signals were also detected in the hyperpallium and the entire DVR, including the arcopallium (Fig. 4a–f,A12.6-A7.4). The regions in which we detected strong signals appeared to correspond to a part of the intercalated nidopallium, which Jarvis *et al*. proposed to be a distinctive and continuous formation of the entopallium, basorostralis, and Field L^26^. Strong *NEFH* expression was restricted to the basorostralis and entopallium, but was not found in the Field L.

### TOX expression in the telencephalon of chick

**Figure 5:**
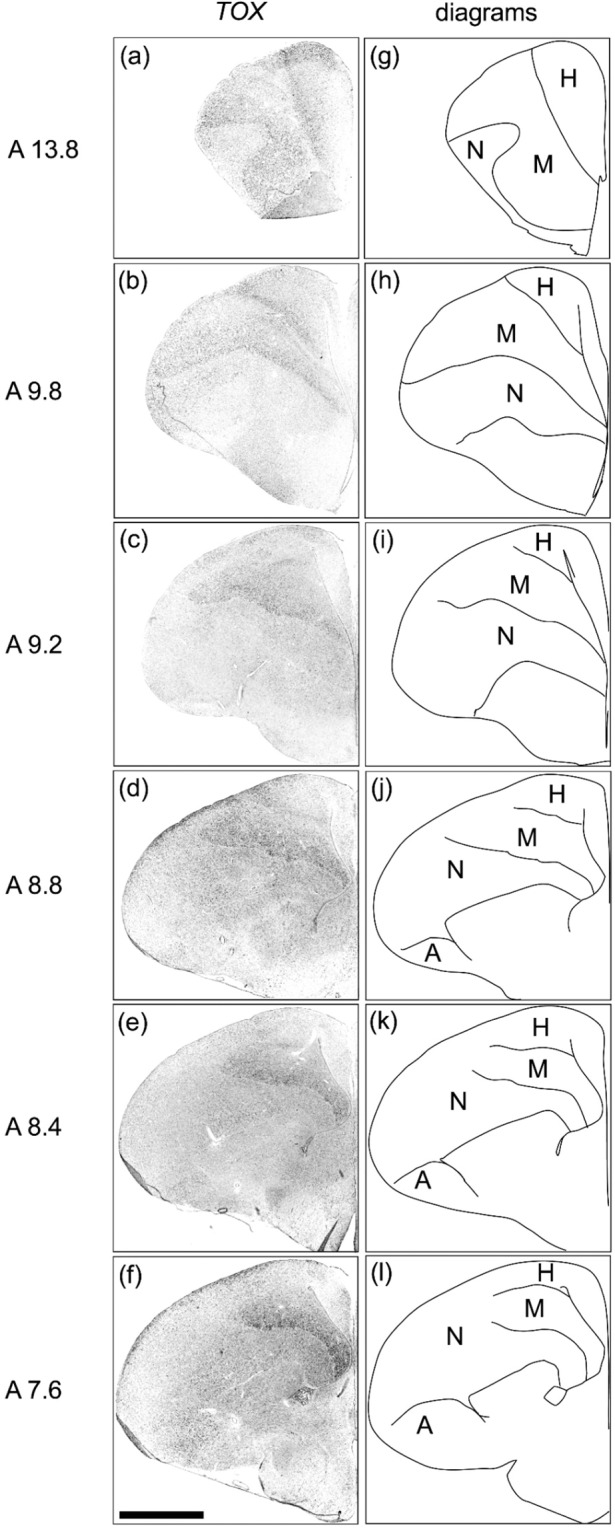
*In situ* hybridisation of *TOX* in P1 chick brains. DIG-labelled RNA antisense. (**a**–**f**) *TOX* probe was used for *in situ* hybridisation in P1 chick brain coronal sections. For *TOX*, sections of three chicks were analysed and representative images of two chick brain sections are shown. (**g**–**l**) Diagrams of coronal sections are shown on the right panels. The levels of the sections (A13.8 to A7.6) correspond to those of the chick atlas by Kuenzel and Masson^50^. A, arcopallium; H, hyperpallium; M, mesopallium; N, nidopallium. Scale bar = 2.5 mm.

Strong signals were detected in the mesopallium (Fig. 5a–f,A13.8-A7.6). In addition, relatively weak signals were detected in the hyperpallium (Fig. 5a–f,A13.8-A7.6) and arcopallium (Fig. 5d–f,A8.8-A7.6).

### CTIP2 and FOXP2 expressions in the telencephalon of chick

**Figure 6:**
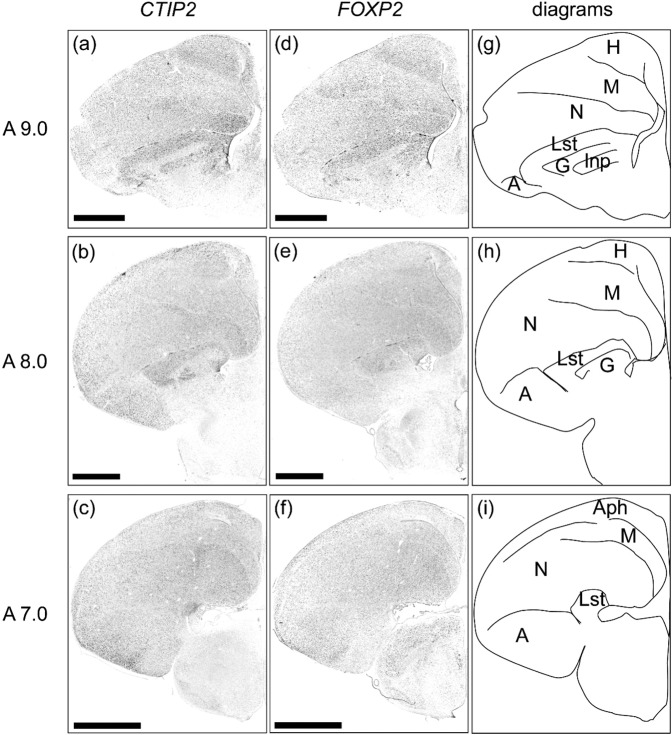
*In situ* hybridisation of *CTIP2* and *FOXP2* in P1 chick brains. DIG-labelled RNA antisense (**a**–**c**) *CTIP2* and (**d**–**f**) *FOXP2* probes for *in situ* hybridisation in P1 chick brain coronal sections. For *CTIP2* and *FOXP2*, sections of four chicks were analysed, and representative images of three chick brain sections are shown. (**g**–**i**) Diagrams of coronal sections are shown on the right panels. The levels of the sections (A9.0 to A7.0) correspond to those of the chick atlas by Kuenzel and Masson^50^. A, arcopallium; Aph, area parahippocampalis; G, globus pallidus; H, hyperpallium; Ins, intrapeduncular nucleus; Lst, lateral striatum; M, mesopallium; N, nidopallium. Scale bar = 2.5 mm.

We further examined expression patterns of the chick orthologue of mammalian neocortical deep layer markers, *CTIP2* and *FOXP2*, in the chick telencephalon. As for *CTIP2*, strong signals were detected in the lateral striatum (LSt), intrapeduncular nucleus (INP), and nidopallium (Fig. 6a,A9.0), and LSt and nidopallium (Fig. 6b,c,A8.0. A7.0). In addition, weak signals were detected in the hyperpallium and the entire DVR (Fig. 6a–c,A9.0-A7.0). As for *FOXP2*, a strong signal was detected in the LSt (Fig. 6d,e,A9.0,A8.0). Weak signals were also detected in the hyperpallium and the entire DVR (Fig. 6d–f).

## Discussion

**Figure 7:**
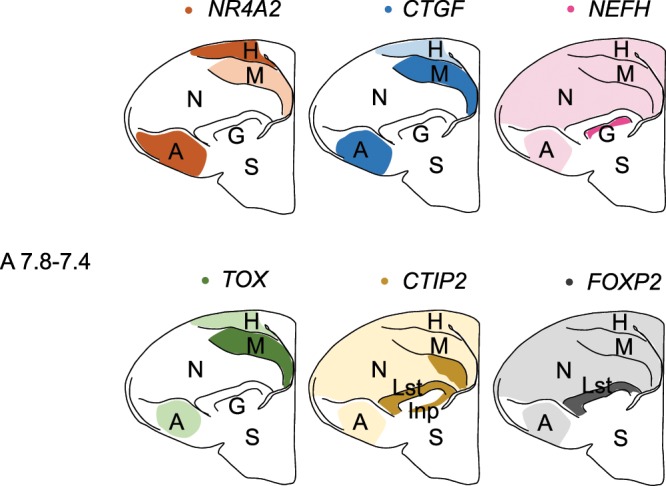
Schematic summary of the expression patterns of the 6 chick orthologues for mammalian L5/6-specific genes (*NR4A2*, *CTGF*, *NEFH, TOX, CTIP2, FOXP2*) in P1 chicks. The representative expression patterns at the levels of sections around A7.8-A7.4 are shown by coloured areas (orange, *NR4A2*; blue, *CTGF*; magenta, *NEFH*; green, *TOX*; yellow, *CTIP2*; grey, *FOXP2*). The darker colours indicate a higher level of gene expression. The levels of the sections correspond to those of the chick atlas by Kuenzel and Masson^50^. A, arcopallium; B, basorostralis; E, entopallium; G, globus pallidus; H, hyperpallium; Inp, intrapeduncular nucleus; Lst, lateral striatum; M, mesopallium; N, nidopallium; S, striatum.

One feature shared by the avian arcopallium and mammalian neocortex L5/6 consists of the neural connections of motor output neurons projecting to sub-pallial targets^5–12^. In this study, we found that chick orthologous genes of mammalian neocortical L5/6 markers, *NR4A2* and *CTGF*, were strongly expressed in a neuronal population of the arcopallium (Fig. 7). Thus, the expression of *NR4A2* and *CTGF* orthologues in the arcopallium could reflect the neural connections in terms of motor output projection between birds and mammals. In contrast, we also found that chick orthologous genes of mammalian neocortical L5/6 markers, *TOX* and *NEFH*, were strongly expressed in regions other than the arcopallium (Fig. 7), which suggests that the expression pattern of these two genes does not reflect the neural connections in terms of motor output. In addition, *CTIP2* was strongly expressed in regions other than the arcopallium and *FOXP2* was expressed in the entire pallium (Fig. 7). The expression of chick orthologues of L5/6 genes did not always support the cell-type homology hypothesis, which suggests that the cell types of brainstem projection neurons between the avian arcopallium and the neocortex L5/6 were not conserved. The genes we used are also expressed in other part of pallium in mammals. However, those genes are basically not expressed together outside of neocortical layers. Neocortical deep layers are the only pallial regions in which all six genes we studied are expressed together (Table S1). Therefore, if the cell-type of brainstem projection neurons in arcopallium and those in deep layer L5/6 are homologous, all six chick orthologues for deep layer markers should be expressed in the arcopallium. Contrary to this assumption, our results showed that two of them were majorly expressed in the arcopallium, while the other genes were not. Thus, not all expression patterns of chick orthologues of L5/6 genes support the cell-type homology hypothesis.

In birds, the arcopallium has motor output neurons that project to sub-pallial targets and is considered to be involved in motor control^27–29^. For example, arcopallium-ablated chicks exhibit less approach behaviours to the imprinting object during filial imprinting^30^, which suggests that the arcopallium is involved in enhancing the subject’s motivation for approach behaviour and/or locomotor activity. We previously found that *NR4A2* was upregulated, accompanying filial imprinting, using cDNA microarray and quantitative RT-PCR^31^. NR4A2 is a transcription factor activated by several signalling cascades^32^. It is possible that NR4A2 modulates arcopallium activity and increases the subject’s motivation for approach behaviour and/or locomotor activity during filial imprinting.

CTGF is a secreted protein which belongs to the Cyr61/CTGF/NOV (CCN) protein family. It binds to and modulates the activity of other growth factors, including insulin-like growth factor, transforming growth factor ß, and bone morphogenetic proteins^33–35^. One study found that CCNs in the nervous system are involved in neuroprecursor proliferation, neuronal survival, and differentiation in mice^35^. Recently, Khodosevich *et al*. found that changes in *CTGF* expression levels in the olfactory bulb led to modifications in local neuronal circuitry and olfactory behaviours^36^. It is possible that CTGF modulates the activity of efferent neurons in the arcopallium and increases the subject’s motivation for approach behaviours or locomotor activity during filial imprinting.

*NEFH* encodes one of the neurofilament triplet components. The level of neurofilament protein has been associated with the extent of neuronal cell myelination^37^, whereby *NEFH*-positive cells are a more heavily myelinated neuronal population. We found that the major expression regions for *NEFH* were the basorostralis and entopallium in the DVR and globus pallidus. The major expression regions for *NEFH* appeared to correspond to a part of the intercalated nidopallium, which supports previous work from Jarvis *et al*.^26^. Those authors found that the intercalated nidopallium receives sensory projections from the thalamus out of the pallium^26^, which suggests that *NEFH* is involved in roles in the cells that have input projection from the thalamus. It is likely that in chicks, *NEFH*-positive cells are a highly myelinated neuronal population within which projection neurons with long dendrites can be found.

TOX is a multifunctional transcription factor involved in corticogenesis via the promotion of neurite outgrowth and regulating the fate of newborn neurons in mouse embryos^20,38^. *TOX* is expressed in the thymus, liver, and brain, and has been studied on the role in lymphocyte development of mice^39^. We found that *TOX* was primarily expressed in the mesopallium in chicks (Fig. 7), which is not known to have the characteristic projections to sub-pallial targets^40–42^. We think that *TOX* is likely to be involved in roles such as neurite outgrowth in neurons within the telencephalon.

The arcopallium is a heterogeneous organisation that consists of a somato-motor region and a limbic region^27–29^. We are beginning to understand which subregions of the arcopallium are related to motor or limbic functions. Recently, one study that used an anterograde tracer found that the lateral arcopallium had characteristic projections to the hippocampus and septum, as well as wide areas of limbic nuclei in the hypothalamus and medial areas of the striatum^11^. These results suggest that the lateral arcopallium is involved in emotion-related behaviours. In addition, diverse projections to midbrain areas were found to derive from the medial arcopallium region^40^. The *NR4A2* and *CTGF* expression patterns found in the present study indicate that the cell populations in the lateral arcopallium were *NR4A2*^+^/*CTGF*^−^ and that those in the medial arcopallium were *NR4A2*^+^/*CTGF*^+^ (Fig. 3). Therefore, a combination of *NR4A2* and *CTGF* expression could be used to precisely characterise cell populations in the subregions of the arcopallium in the chick brain.

Much like the mammalian L5/6, the hyperpallium also has motor output neurons projecting to the spinal cord or thalamus^5,6,12^. All six orthologues for L5/6-specific genes used in this study were expressed in neural populations of the hyperpallium of chicks (Fig. 7), which shows that the expression patterns reflected the neural connections patterns in terms of motor output projections to sub-pallial targets. Given that the avian dorsal pallium derivative region (hyperpallium) is considered to be homologous to the mammalian neocortex^43,44^, this is reasonable.

The mesopallium is not known to have the characteristic projections to sub- pallial targets. The major projections from the mesopallium are distributed within the telencephalon, and the mesopallium has strong reciprocal fibre connections with regions in the nidopallium^40–42^. We found that all six orthologous genes used in this study were expressed in the mesopallium (Fig. 7). This suggests that, in the mesopallium, these six genes were expressed in neurons projecting within the telencephalon rather than in neurons projecting to the spinal cord out of the telencephalon. In the case of *NR4A2*, mouse *Nr4a2* has been reported to be expressed not only in the neocortex, but also in other parts of the pallium (the claustrum)^25^. Furthermore, one study reported that an early *NR4A2*-positive population within the chick mesopallium represents the lateropallial claustrum homologue in mouse embryos^25^. It is important to consider the gene expression patterns in other parts of the pallium, such as the claustrum, together with a hodological analysis of connections to compare the gene expression patterns in the mammalian cortex and in birds’ DVR.

Analysing homologies through developmental origins is useful to understand the evolution of brain complexities between mammals and birds. As mammalian neocortex derives from the dorsal pallium, while the DVR of birds derives from the lateroventral pallium^43,44^, the neocortex and DVR are not homologous in terms of developmental-based homology. In contrast, the mammalian brain regions derived from the lateroventral pallium such as the amygdala, claustrum and dorsal endopiriform nucleus might be homologous to the parts of the birds’ DVR in terms of developmental-based homology. For example, the whereabouts of the avian pallial amygdala remain uncertain, but there are several studies suggesting that a part of the caudal DVR, including at least the caudal nidopallium and the whole arcopallium of birds, may be avian pallial amygdala^43–45^. As for the claustrum, a recent study suggested that a part of the avian lateral mesopallium (superficial mesopallial cortical structure) is the strict homologous region of the mammalian claustrum^25,44,46^. A part of lateral nidopallium is the homologous region of mammalian dorsal endopiriform nucleus^25,46,47^.

We found that chick *NR4A2* was majorly expressed in the arcopallium (Fig. 7) which was suggested to be avian pallial amygdala, whereas mouse *Nr4a2* was not expressed in the pallial amygdala during embryonic development nor in adults^24,25^. This evidence showed that chick *NR4A2* expression in the arcopallium did not reflect developmental-based homologies. Similarly, we also found that chick *CTGF* was majorly expressed in the arcopallium (Fig. 7) whereas mouse *Ctgf* was not expressed in the pallial amygdala (P8 and adult)^24^. This evidence showed that chick *CTGF* expression in the arcopallium did not reflect developmental-based homologies. One feature shared by the avian arcopallium and mammalian neocortex L5/6 consists of the neural connections of motor output neurons projecting to sub-pallial targets. We assume that the similarity in the expression pattern might better reflect function than homology and be the result of convergent evolution. Chick *TOX* were majorly expressed in the medial mesopallium (Fig. 7), which was not the avian homologous region of mouse *Tox* expressing domains (the hippocampus and claustrum, Table S1). Chick *NEFH* were expressed in the basorostralis (Fig. 4a,b) and entopallium (Fig. 4c,d). All of these *NEFH*-expressing domains were not the avian homologous region of mouse *Nefh*-expressing domains (hippocampus, piriform cortex and olfactory bulb, Table S1).

In this study, we only tested a small number of genes. However, our results suggested that avian hyperpallium, which share developmental-based homology and functional-based analogy between birds and mammals, displayed conserved expression patterns of neocortical deep layer genes (Fig. 7). In contrast, our results suggest that most pallial regions have undergone major reorganisation in terms of gene expression patterns between birds and mammals. This is consistent with the findings of recent comprehensive transcriptome analyses^14,15^, which have demonstrated that gene expression patterns in the adult mouse cortex are not compatible with those of the adult chick pallium. Over hundreds of millions of years, pallium of birds and mammals has become astonishingly diversified.

## Conclusion

The avian arcopallium and mammalian neocortex L5/6 share the feature of neural connections of motor output neurons projecting to sub-pallial targets. We used chick orthologues of mammalian neocortical L5/6 markers and performed *in situ* hybridisation in the chick telencephalon. We found that *NR4A2* and *CTGF* expression reflected the neural connection patterns between avian DVR and mammalian neocortex, but *TOX, NEFH, CTIP2 and FOXP2* expression patterns did not. Thus, not all the expression patterns of chick orthologues of L5/6 genes support the cell-type homology hypothesis. This suggests that the cell types of brainstem projection neurons are not conserved between the avian arcopallium and the mammalian neocortex L5/6.

## Methods

### Animals and tissues

Fertilized eggs of domestic chicks (*Gallus domesticus*, the Cobb strain) were purchased from a local dealer (3-M, Aichi, Japan) and incubated at Teikyo University (Kaga, Itabashi-ku, Tokyo). Animal experiments were carried out as described previously^48,49^. Briefly, newly hatched chicks (P0) were captured and placed in dark plastic enclosures in a breeder at 30 °C for one day (P1). P1 chicks were deeply anesthetized with a 1:1 mixture solution of ketamine (10 mg/ml, ketalar-10, Sankyo Co., Tokyo, Japan) and xylazine (2 mg/ml, Sigma, St. Louis, Missouri, USA) by intraperitoneal injection (0.40 ml/individual) and perfused through the heart with 4% paraformaldehyde in 0.1 M phosphate buffered saline (pH 7.5) (PFA-PBS). In this study, we used 2 chicks for the *NR4A2* condition, 3 for the *CTGF* condition, 4 for the *NEFH* condition, 3 for the *TOX* condition, and 4 for the *CTIP2* and *FOXP2* conditions (a total of 7 chicks). Dissected brains were immersed in PFA-PBS overnight at 4 °C and placed in an 18% sucrose/PFA-PBS solution for cryoprotection for two days at 4 °C. Next, brains were embedded in Tissue-Tek OCT compound (Sakura Finetechnical, Tokyo, Japan), frozen immediately on dry ice, and stored at −80 °C until use. All procedures were reviewed and approved by the committee on animal experiments of Teikyo University and conducted under the guidelines of the national regulations for animal welfare in Japan.

### cDNA cloning and RNA probe preparation

For preparation of probes, total RNA was extracted from the chick brain using TRIzol Reagent (Invitrogen, Carlsbad, CA, USA) and reverse-transcribed with Super-Script III (Invitrogen, Carlsbad, CA, USA) using an oligo (dT) primer, according to the manufacture’s protocol. RT-PCR was performed using the following gene specific primer pairs: 5′-atgaggctcccaagaaggat-3′ and 5′-aagcgatcggaacataccac-3′ for *NEFH*; 5′-tgcctggacccctactattg-3′ and 5′-tggactgaactggatggtga-3′ for *TOX*; 5′-ttccggttaagcagacgaag-3′ and 5′-ggaatgtggacggtgcttac-3′ for *NR4A2*; 5′-tttgtctactgaccccaaacagt-3′ and 5′-caaagcattacacataggcacaa-3′ for *CTGF*; 5′-agaccgtcttctcacgccta-3′ and 5′-gaactgtttcctgccagctc-3′ for *CTIP2*; 5′-gtctccccagcagctacaag-3′ and 5′-ggtggtgatgctttggaagt-3′ as forward and reverse primers for *FOXP2*, respectively. PCR products were subcloned into pGEM-T easy vector (Promega, Madison, WI, USA), Sanger sequenced, and confirmed. Plasmids containing the cDNA fragment for *NR4A2*, *CTGF*, *NEFH*, *TOX, CTIP2*, and *FOXP2* were amplified by PCR with an M13 primer pair. The amplicons containing the T7 and SP6 promoter sites were purified using a PCR purification kit (Qiagen, Valencia, CA, USA). The digoxigenin (DIG)-labelled sense and antisense RNA probes were prepared by *in vitro* transcription using a DIG RNA labelling kit (Roche, NJ).

### *In situ* hybridisation

The frozen brain blocks were cut into 18 µm-thick sections using a cryostat (Leica CM3050S or Leica CM1850, Leica Biosystems, Nußloch, Germany). Serial coronal sections were prepared from a level A 13.8 to A 7.0 of the Kuenzel and Masson’s atlas^50^. *In situ* hybridisation was performed as described previously with some modifications^51^. Briefly, brain sections were fixed in 4% PFA-PBS, pretreated, and hybridised with DIG-labelled riboprobes at 60 °C. After stringent washes, DIG-labelled riboprobes were detected immunocytochemically with alkaline phosphatase-conjugated anti-DIG antibody (1:1,000; Roche, NJ). To visualise the signals, chromogenic reaction with a nitro blue tetrazolium/5-bromo-4-chloro-3-indolyl phosphate were performed at room temperature for following hours: *NR4A2*, 24 hours; *CTGF*, 37–39 hours; *NEFH*, 12.5–19 hours; *TOX*, 37–39 hours; *CTIP2*, 36–38 hours and *FOXP2*, 46–48 hours. In every experiment, sense probes were used as negative controls.

### Imaging

Digital images of sections were obtained with NanoZoomer 2.0HT or NanoZoomer XR systems (Hamamatsu Photonics, Shizuoka, Japan) and microscopic fields of interest were cropped using NDP.view2 software (Hamamatsu Photonics, Shizuoka, Japan). The images were then converted to 8-bit and the brightness and contrast of images was adjusted using ImageJ (https://imagej.nih.gov/ij/).

## Supplementary information

Supplementary Information.

## Data Availability

The datasets generated during and/or analysed during the current study are available from the corresponding author on reasonable request.
