# Supplementary material for: The chick pallium displays divergent expression patterns of chick orthologues of mammalian neocortical deep layer-specific genes
Source: Sci Rep. 2019 Dec 31;9:20400. doi: 10.1038/s41598-019-56960-4 (PMC6938507; doi:10.1038/s41598-019-56960-4)
Supplement: Supplementary file 1 — Supplementary Information. [file 41598_2019_56960_MOESM1_ESM.pdf]

Supplementary information to

The chick pallium displays divergent expression patterns of chick orthologues of mammalian neocortical deep layer-specific genes

Toshiyuki Fujita[1], Naoya Aoki[1], Eiko Fujita[1], Toshiya Matsushima[2], Koichi J. Homma[1], and Shinji Yamaguchi[1]*

[1] Faculty of Pharmaceutical Sciences, Department of Life and Health Sciences, Teikyo University, 2-11-1 Kaga, Itabashi-ku, Tokyo, 173-8605, Japan
[2] Department of Biology, Faculty of Science, Hokkaido University, Hokkaido 060-0810, Japan

*Corresponding author:
Shinji Yamaguchi, Ph.D.
E-mail: shinji-y@pharm.teikyo-u.ac.jp
Tel: +81-3-3964-8136
Fax: +81-3-3964-8415

Supplementary Table S1

Supplemental Table S1. Pallial expression of the cortical deep layer-selective genes in mice

Gene name	Cortical deep layers (L5 and/or L6)	Claustrum	Endopiriform nuclei	Piriform cortex	Pallial amygdala	Hippocampal formation	Olfactory bulb	References[1)]
Nr4a2	+	+	+	-	-	+	+	Wang, et al., 2011, Allen Brain Atlas[2)]
Ctgf	+	-	+	-	-	-	+	Wang, et al., 2011, Allen Brain Atlas[3)]
Nefh	+	-	-	+	-	+	+	Allen Brain Atlas[4)]
Tox	+	+	-	-	-	+	-	Allen Brain Atlas[5)]
Ctip2	+	-	-	+	+	+	+	Allen Brain Atlas[6)]
Foxp2	+	-	-	+	-	-	+	Allen Brain Atlas[7)]

1) Allen Mouse Brain Atlas's reference plate #12 for cortical deep layers, claustrum, endopriform nuclei, piriform cortex, hippocampal formation and olfactory bulb, and #3 for pallial amygdala in Allen Brain Atlas
2) Experiment #733 in Allen Brain Atlas
3) Experiment #1184 in Allen Brain Atlas
4) Experiment #71920442 in Allen Brain Atlas
5) Experiment #69817025 in Allen Brain Atlas
6) Experiment #74990505 in Allen Brain Atlas
7) Experiment #68918282 in Allen Brain Atlas
